# Milk and Dairy Products: Exploring Production, Processing, and Quality Control

**DOI:** 10.3390/foods15091571

**Published:** 2026-05-02

**Authors:** Christopher Pillidge, Jayani Chandrapala, Mayumi Silva

**Affiliations:** School of Science, RMIT University, Melbourne, VIC 3083, Australia; mayumi.silva@rmit.edu.au

## 1. Introduction

Milk quality control primarily involves testing milk for its microbiological, physical, and chemical properties during both on-farm collection and transport and at the factory. Dairy products are subsequently monitored for their safety and quality after processing and distribution [1,2,3]. This monitoring often involves independent food testing laboratories. While such testing is essential, the determinants of milk production, processing, and dairy product quality control are broader and involve many different unrelated factors and interactions.

Understanding how these factors combine to affect milk and dairy product quality can be difficult. Of course, some contributing factors are obvious—on-farm hygiene or milk chemical composition, for example—but others are less so (for example, the effect of climate change [4]). This challenge is especially difficult given the complexity of the food supply chain. Not only that, but milk itself is a complex biological fluid, typically comprising water (~87%), lactose, lipids, proteins, minerals, enzymes, minor components, and bioactive compounds [5]. As such, it is highly vulnerable to microbiological and chemical deterioration. Deficiencies at any stage in collection and processing compromise safety, shelf life, product quality, and dairy product functionality [6]. Therefore, understanding the determinants of milk and dairy product quality has been the subject of much ongoing research.

It is impossible to encapsulate in one Special Issue all of the factors that fall within the scope of this title. Instead, the editors have selected five papers from around the world on different aspects of milk and dairy product quality control that represent the entire scope of ‘farm to table’. These papers will be of much interest to the general Foods audience, especially those in the dairy sector.

## 2. Overview of the Published Articles

The aim of this section is to introduce each of the five contributions to pique the audience’s interest. The first paper describes a protective LAB strain, *Lacticaseibacillus casei* VC201, applied to a PDO cheese, namely Valtellina Casera from Northern Italy. While the use of starter (acidifying), adjunct (flavour), and probiotic (health) cultures continues to be of importance to the worldwide dairy industry, along with supporting research to continually develop new strains, increasing attention is being given to the concept of protective cultures for dairy. These cultures have been used for many years in the meat industry, where bacterial strains such as coagulase-negative staphylococci or LAB either alone or in combination are applied to inhibit spoilage and/or pathogenic bacteria, through the production of inhibitory organic acids, H_2_O_2_, and/or various bacteriocins [7]. The idea of using protective cultures in dairy products is more recent and fits within the scheme of natural approaches to ensure product safety and quality while reducing the need for chemical additives that can upset product sensory and nutritional properties [8,9]. The first paper, by Bonazza et al., describes the screening of 34 LAB strains isolated from Bitto and Valtellina Casera PDO cheeses for their anti-*Clostridium* activity. It has been known for many decades that *Clostridium* spores (especially *C. tyrobutyricum*) can be a problem in certain hard cheeses since they survive pasteurization and germinate during ripening, thereby producing unwanted gas and off-flavours, including those associated with excess butyric acid (late-blowing defect) [10]. One LAB strain, VC201, was isolated and found to be highly effective through its production of organic acids. Moreover, this strain had additional utility in that it also improved the cheese flavour profile, which was assessed in the laboratory and by a sensory panel. Research leading to the discovery of useful strains like VC201 benefits not only commercial culture companies but also the entire dairy industry.

The second contribution, by Yang et al. from Qinghai Province, China (near the Tibetan Plateau), describes risk reduction and quality control in camel milk collection. Camel milk differs from cow milk, with 25% less fat and lactose, no ϐ-lactoglobulin (making it less allergenic), and different ϐ-caseins aiding in digestibility. It is also higher in minerals, immunoglobulins, and vitamins [11,12]. However, the milk yield per camel is lower than that of cows, and there is greater reliance on small-scale farming. Most camel milk production occurs in Africa (Kenya and Somalia), but Xinjiang, Inner Mongolia, and Gansu Provinces in Western China are also significant producers, supplying the increasing demand for camel milk in Asia [13,14]. Yang et al. describe quality-related factors that apply to camel milk collection, particularly in small-scale production; these factors (milk adulteration, microbial contamination, and nutritional variability) are equally important in bovine milk collection. In this study, some 80 camel milk samples were analysed, and it was found that adulteration with cow milk was a significant issue, along with microbial contamination. Problems occurred at a higher rate when hand-milking was practised versus machine-milking. A recent review [15] indicates that the problem of milk adulteration is common in many parts of the world. The combination of rapid diagnostic tools (such as DNA testing) has provided many new insights and tools to help with this problem. The authors of the present study proposed a set of culturally adaptive quality control measures to address these issues.

The third contribution, by Zhang et al., describes genetic markers affecting the milk quality of domestic yak herds from a nearby region (Gansu Province). Domestic yaks (*Bos grunniens*) and cows (*Bos taurus*/*Bos indicus*) are closely related species within the genus *Bos* that may be crossbred. Such crossbreeding (and back-crossing) has meant that gene introgression from cattle into yaks has occurred over many thousands of years (indeed, 686 cattle genes have been identified in the yak genome [16]). Also, in comparison to cows, yaks are more suited to high-altitude farming. Because of the importance of yak milk to Central Asia, genetic factors affecting yak milk quality are being studied. Just as genomic herd testing combined with breeding and data-driven decision making has led to improvements for the traditional dairy industry, the same is true for yaks. In all mammalian species, specific gene polymorphisms control key milk properties like protein composition, with two examples being A1 versus A2 ϐ-casein [17] and milk production (lactation) [18]. In their paper, Zhang et al. investigate the correlation between various single-nucleotide polymorphisms (SNPs) in two yak genes (*IQGAP2* and *CRTAC1*, whose properties they describe), aiming to identify potential marker loci that can be used for enhancing milk quality. Using the Illumina Yak cGPS 7K liquid chip, they genotyped 162 yaks and identified five SNPs. Following association analyses, the authors identified three key milk quality traits that were impacted, namely milk fat, lactose, and total solids content. Studies like these underpin yak breeding strategies in much the same way that genetic studies on cows have underpinned commercial cow breeding programs.

The fourth contribution, by Poo et al. (Chile), considers laboratory testing of milk: in particular, how can milk quality tests carried out by different laboratories be verified and their applied methods guaranteed for accuracy and reliability? The answer lies in proficiency testing (PT), which allows interlaboratory comparisons using a standard method and certified reference materials (CRMs). PT is performed by accreditation authorities worldwide for a wide range of physical, chemical, and microbiological tests (in Australia, the leading accreditation body is the National Association of Testing Authorities or NATA). Like many dairy-producing countries, Chile relies on thousands of producers that provide dairy products to its population. Poo et al. describe eight PT rounds dedicated to the determination of fat and crude protein by mid-IR spectroscopy using a standardised method (ISO 8196-3) [19] of raw milk samples obtained from several producers in Southern Chile, using CRMs from a German company. The PT was done by the Metrology Division of the LACM^®^ (Laboratory for Measurement Quality Assurance of the Austral University of Chile), with variations to the number of samples and analyses made according to Standard ISO 13528:2022 [20]. Results showed that the assigned values for the CRMs were mostly consistent with results obtained by the various participants, with the PT being carried out over a four-year period (2017 to 2021). It was concluded that the study design for the assignment of the reference value was appropriate for PT with a small number of participants. The authors further recommended that the SD for the testing be updated from 0.075 g/100 g milk to 0.053 g/100 g milk.

The fifth contribution, by Wijegunawardhana et al., is a review on heat-induced component interactions that occur during the manufacture of milk–tea powders. These are made by combining dried powders or liquid milk, depending on the manufacturing process, with other added ingredients—such as maltodextrin, fructans, galacto-oligosaccharides, lactose, and glucose polymers—and tea. These ingredients are added to many powdered milk products to optimize their physiochemical, nutritional, and sensorial properties [21]. Variations in these ingredients and the processing method (dry- or wet-mixing) result in different molecular interactions. These interactions include protein–protein, protein–carbohydrate, protein–polyphenol, and protein–carbohydrate–polyphenol conjugations, among others, and are especially important during wet-mixing, since that process involves heating, concentration, and spray drying. This review considers these interactions at the molecular level in the context of milk–tea powders. Apart from the milk ingredients listed, tea contains additional components like polyphenol flavonoids and small amounts of carbohydrates, proteins, vitamins, and minerals. The aforementioned interactions are reviewed in detail, providing a basis to modify formulations and processing steps and thereby the means to improve product properties and storage stability. A recent estimate suggests that the global milk–tea powder market will be worth well over USD 4 billion in 2026, with the Asia–Pacific Region having the greatest market share [22].

## 3. Conclusions

Dairy processing and milk quality issues have received considerable attention, not only by scientific establishments but also by dairy companies and food regulatory agencies, and this is reflected in a wide body of the existing literature. Most of this attention has been within a ‘traditional’ dairy context, that is, concerning bovine, caprine, or ovine milks and their production and associated dairy farming practices and dairy product manufacturing.

Two contributions to this Foods Special Issue provide a somewhat different perspective, focusing on less common milk-producing animals (camels and yaks). At the same time, within this collection, the familiar milk quality themes of microbial spoilage, on-farm collection practices, herd genetics and breeding, laboratory testing verification, and product component interactions are considered, making this Special Issue of considerable interest to a wider Foods audience. The editors consider that these papers will be a useful addition to our scientific knowledge on these topics.

## References

[1] Castellini G., Barello S., Bosio A.C. (2023). Milk quality conceptualization: A systematic review of consumers’, farmers’, and processing experts’ views. Foods.

[2] Martin N.H., Evanowski R.L., Wiedmann M. (2022). Invited review: Redefining raw milk quality—Evaluation of raw milk microbiological parameters to ensure high-quality processed dairy products. J. Dairy Sci..

[3] Srivastava A., Sharma S., Sahu J.K., Chandra Deka S., Nickhil C., Haghi A.K. (2025). Quality assurance and control in food and dairy products. Engineering Solutions for Sustainable Food and Dairy Production.

[4] de São José A.N., Ferrarez Bouzada Viana M.C., Machado M.G.P., de Morais A.S.L., do Carmo Resende U., de Oliveira Mota H., Júnior M.L.P., da Rocha A.F., de Carvalho D.O., de Jesus Hartmann Nader P. (2025). Global warming impacts on lactating mammalian milk: A systematic review. Sci. Total Environ..

[5] Acquavia M.A., Villone A., Rubino R., Bianco G. (2025). A comprehensive review of milk components: Recent developments on extraction and analysis methods. Molecules.

[6] Formaggioni P., Franceschi P. (2024). New insights into milk and dairy products: Quality and sustainability. Foods.

[7] Jůzl M., Kalhotka L., Kameník J., Dušková M., Ondruchová S., Slováček J. (2026). Protective cultures applied in meat products: Technological functions, safety aspects and current advances: A review. Processes.

[8] Silva C.C.G., Silva S.P.M., Ribeiro S.C. (2018). Application of bacteriocins and protective cultures in dairy food preservation. Front. Microbiol..

[9] Bintsis T., Papademas P. (2024). The application of protective cultures in cheese: A review. Fermentation.

[10] Velasco R., Cabeza M.C., Ordóñez J.A. (2025). Current approaches to minimize the late blowing defect of cheese. Cogent Food Agric..

[11] Almasri R.S., Bedir A.S., Ranneh Y.K., El-Tarabily K.A., Al Raish S.M. (2024). Benefits of camel milk over cow and goat milk for infant and adult health in fighting chronic diseases: A review. Nutrients.

[12] Ho T.M., Zou Z., Bansal N. (2022). Camel milk: A review of its nutritional value, heat stability, and potential food products. Food Res. Int..

[13] El Agamy E.I., Park Y.W., Haenlein F.W. (2006). Camel milk. Handbook of Non-Bovine Mammals.

[14] El-Hanafy A.A., Saad Y.M., Alkarim S.A., Almehdar H.A., Alzahrani F.M., Almatry M.A., Uversky V.N., Redwan E.M. (2023). Yield and composition variations of the milk from different camel breeds in Saudi Arabia. Sci.

[15] Khomane V., Kamble D., Lokhande A., Patil B., Lashkare S., Kubade K., Khobragade S. (2024). A comprehensive review on adulteration of milk. Int. J. Adv. Biochem. Res..

[16] Liu X., Liu W., Lenstra J.A., Zheng Z., Wu X., Yang J., Li B., Yang Y., Qiu Q., Liu H. (2023). Evolutionary origin of genomic structural variations in domestic yaks. Nat. Commun..

[17] O’Callaghan T. (2020). An overview of the A1/A2 milk hypothesis. Dairy Nutr. Forum.

[18] Cole J.B., Makanjuola B.O., Rochus C.M., van Staaveren N., Baes C. (2023). The effects of breeding and selection on lactation in dairy cattle. Anim. Front..

[19] (2022). Milk—Definition and Evaluation of the Overall Accuracy of Alternative Methods of Milk Analysis. Part 3: Protocol for the Evaluation and Validation of Alternative Quantitative Methods of Milk Analysis.

[20] (2022). Statistical Methods for Use in Proficiency Testing by Interlaboratory Comparison.

[21] Soni S., Anvil J.W., Kurian C., Chakraborty P., Paari K.A. (2024). Food additives and contaminants in infant foods: A critical review of their health risk, trends and recent developments. Food Prod. Process. Nutr..

[22] Gore A. (2026). Milk Tea Powder Market Analysis 2026.

